# Expression of Hepcidin and Ferroportin in the Placenta, and Ferritin and Transferrin Receptor 1 Levels in Maternal and Umbilical Cord Blood in Pregnant Women with and without Gestational Diabetes

**DOI:** 10.3390/ijerph13080766

**Published:** 2016-07-28

**Authors:** Anqiang Yang, Jun Zhao, Minhua Lu, Ying Gu, Yunlong Zhu, Daozhen Chen, Jinyan Fu

**Affiliations:** 1 Department of Pathology, Wuxi Maternity and Child Health Hospital Affiliated to Nanjing Medical University, Wuxi 214002, China; yaq1234@126.com (A.Y.); mhlu2005@sina.com (M.L.); 2 Clinical Laboratory, Wuxi Maternity and Child Health Hospital Affiliated to Nanjing Medical University, Wuxi 214002, China; chalange@163.com (J.Z.); chendaozhen@163.com (D.C.); 3 Department of Obstetrics, Wuxi Maternity and Child Health Hospital Affiliated to Nanjing Medical University, Wuxi 214002, China; 18914137366@189.cn (Y.G.); sequoia113847@163.com (Y.Z.)

**Keywords:** gestational diabetes, placenta, ferritin, transferrin receptor 1, ferroportin

## Abstract

*Background*: Regulation of iron transfer from mother to fetus via the placenta is not fully understood and the relationship between stored iron status in the mothers’ serum and gestational diabetes (GDM) in case–control studies is controversial. The present study aimed to detect circulating soluble transferrin receptor (sTfR) and ferritin levels in maternal and umbilical cord blood. We also examined the expression of hepcidin (Hep), transferrin receptor (TfR1), and ferroportin (FPN) in the placenta in pregnant women with and without GDM at full term. *Methods*: Eighty-two women participated (42 with GDM and 40 without GDM [controls]). Maternal samples were collected at 37–39 weeks’ gestation. Umbilical cord blood was collected at birth. Ferritin and sTfR levels in maternal serum and umbilical cord blood, and Hep, TfR1, and FPN protein expression in plac enta were compared between the GDM and non-GDM groups. Serum ferritin (SF) was measured by electrochemiluminescence assay and sTfR was measured by ELISA. Hep, TfR1, and FPN expression was measured by immunohistochemistry. *Results*: Maternal serum sTfR levels were significantly elevated in the GDM group compared with the non-GDM group (*p* = 0.003). SF levels in cord blood in the GDM group were significantly higher than those in the non-GDM group (*p* = 0.003). However, maternal hemoglobin and SF, and umbilical cord sTfR levels were not different between the groups. In placental tissue, FPN expression was higher and hepcidin expression was lower in the GDM group compared with the non-GDM group (*p* = 0.000 and *p* = 0.044, respectively). There was no significant difference in TfR1 between the groups (*p* = 0.898). *Conclusions*: Women with GDM transport iron more actively than those without GDM at term pregnancy. Maternal iron metabolism in GDM may play a role in fetal/placental iron demand and in the overall outcome of pregnancy.

## 1. Introduction

Gestational diabetes (GDM) is a type of diabetes which is the most common metabolic disorder during pregnancy [1,2]. In a study in Tianjin ,China, a steady 2.8-fold increase in the prevalence of gestational diabetes has been observed from 1999 to 2008 (from 2.4% to 6.8%) [3]. Several investigations have shown that GDM is linked to the complications of macrosomia, birth trauma such as increased maternal lacerations and neonatal shoulder dystocia, and neonatal metabolic disorders such as hypoglycemia and hyperbilirubinemia [4,5,6,7,8] GDM also increases the risk of glucose metabolism disorders and type 2 diabetes in later life in the mother and newborn [9,10].

Recently, some studies have reported that a high iron load and iron metabolism disorders are associated with an increased risk of glucose metabolism disturbances [11,12,13,14]. GDM is associated with several fetal and maternal consequences, therefore, early diagnosis of this common metabolic disorder is important for preventing maternal and prenatal complications. Nevertheless, regulation of iron transfer from mother to fetus via the placenta is not fully understood. Additionally, the relationship between stored iron status in the mothers’ serum and GDM seen in cross-sectional and case-control studies is controversial, therefore, further investigations on these issues are required.

The present study aimed to measure circulating sTfR and SF levels in maternal and umbilical cord blood. This study also aimed to detect expression of major iron proteins, such as HepTfR1, and FPN in the placenta to determine changes in maternal-fetal iron metabolism in pregnant women with and without GDM at term pregnancy. Hep is the central regulator of the iron homeostasis, acting on the iron exporter of the enterocytes and macrophages and suppressing iron uptake and iron release [15]. TfR1 is a sensitive indicator of iron deficiency and FPN is the only known cellular iron exporter [16].

## 2. Materials and Methods

### 2.1. Study Population

The case-control study was conducted at Wuxi Maternity and Child Health Care Hospital of Nanjing Medical University (China) from May 2014 to June 2015. All samples were collected with the approval of the appropriate institutional ethics committee (IORG0001416) and written consent was provided by each pregnant woman. The case history and the complete clinical examination findings of the patients were recorded. GDM was diagnosed based on a 2-h, 75-g oral glucose tolerance test (OGTT) during the second trimester (from 24 to 28 weeks’ gestation). The criterion for diagnosis of GDM was at least one abnormal value of the following: fasting glucose level ≥5.1 mmol/L after an overnight fast; and blood glucose level >10 mM 1 h after or >8.5 mM 2 h after consuming 75 g of glucose. Patients with chronic hypertension, preeclampsia, acute or chronic inflammatory or infective diseases, prior renal disease, liver disease, multiple gestation pregnancy, iron deficiency anemia, maternal age <15 years, congenital or chromosomal abnormalities of the fetus, and pregnant women with a family history of diabetes were excluded from our study. A total of 42 women with GDM-newborn pairs were selected (30 were treated with diet only and 12 with diet and insulin) and 40 pregnant women with normal glucose tolerance (NGT) were recruited as controls. The relevant clinical details of these pregnancies are shown in Table 1.

### 2.2. Measurement and Collection of Samples

SF and sTfR levels were measured at 36–39 weeks of gestation. Whole cord blood obtained at delivery was assayed at that time for hemoglobin levels. The remaining blood was centrifuged, and the serum was separated and frozen at −80 °C for subsequent analysis. Approximately 4–5 mL of venous blood was collected in plain and EDTA containers. Whole blood collected in EDTA tubes was used for hemoglobin (Hb) analysis. Blood collected in plain tubes was centrifuged at 3000 rpm for 5 min to separate serum. Separated serum was transferred to a fresh vial and was used for the estimation of SF and sTfR levels. Placental tissues were obtained immediately after delivery. Fetal membranes were cut into small pieces (1 cm^3^). The fetal surface and maternal surface were collected. Tissue pieces were individually rinsed at 4 °C in sterile diethyl pyrocarbonate-treated phosphate-buffered saline to wash off maternal and fetal blood. Tissue was snap frozen in liquid nitrogen within 5 min after removal, and stored at −80 °C freezer until further analyses. Serum glucose levels were estimated by the glucose oxidase and peroxidase method using a human reagent kit. Blood Hb levels and hematocrit (Hct) were analyzed using a Beckman Coulter LH 750 hematology analyzer (Beckman Coulter, Inc., Brea, CA, USA). Serum ferritin levels (ng/mL) were measured with an electrochemiluminescence assay (E170; Roche, Herts, UK) Levels of sTfR (ng/mL) were assessed with an enzyme-linked immunosorbent assay (ELISA) (sTfR kit; R & D Systems, Inc., Minneapolis, MN, USA).

### 2.3. Immunohistochemistry

Protein expression levels of Hep, TfR1, and FPN in placental tissue were measured by immunohistochemistry in paraffin-embedded sections. Antigen retrieval was performed by treatment with citric acid (pH 6.0) for 15 min. Non-specific antibody binding was blocked by incubating with 10% fetal bovine serum for 20 min. Mouse anti-human hepcidin monoclonal antibody (1:150) and rabbit anti-human TfR1 (1:1000) and ferroportin antibodies (1:25) were added for 2 h at room temperature. Sections were then washed with phosphate-buffered saline and incubated with HRP-polymer anti-mouse/rabbit IgG (MaxvisionTM2 kit; Maxim Bio, Fuzhou, China) for 15 min. The antigen–antibody complexes were visualized using DAB and counterstained with hematoxylin.

### 2.4. Immunohistochemical Evaluation

All slides were examined independently by two pathologists who were blinded to the patients’ clinical data. The staining intensity of TfR, ferroportin, and Hepcidin expression in all specimens was semi-quantitatively scored. Five fields were randomly selected and 15 images of each sample from each group were taken with the microscope settings unaltered (×400 magnification). Staining intensity in syncytiotrophoblast (STB) cells was scored basing on the combination of staining intensity of immunohistochemical images with the percentage of positive cells, using a previously published method [17]. Briefly, no staining was scored as 0, 1%–10% of positively stained cells was scored as 1, 11%–50% as 2, 51%–80% as 3, and 81%–100% as 4. Staining intensity was rated on a scale of 0–3, with 0 = negative, 1 = weak, 2 = moderate, and 3 = strong. The raw data were converted by multiplying the quantity and staining intensity scores. Negative controls were stained without prior incubation with the primary antibody.

### 2.5. Statistical Analysis

Statistical analyses were performed using SPSS for Windows software version 17.0 (SPSS Inc., Chicago, IL, USA). The Kolmogorv-Smirnov method was used to test data distribution normality. Serum ferritin failed the normality test. Therefore, the data were described using median and percentile values. The non-parametric chi-square test was used to compare differences between groups. Parametric data are expressed as mean ± standard deviation (SD) and analyzed by the *t* test.

## 3. Results

### 3.1. Sample Clinical Characteristics

Eighty-two women participated in the study, with 42 in the GDM group and 40 in the non-GDM group (controls). Newborn birth weight, gestational age, gestational weeks and ethnicity were not different between the groups (as Table 1).

### 3.2. Maternal and Cord Blood Iron Metabolism Biochemistry

At full term, women in the GDM group had significantly higher sTfR levels than did those in the non-GDM group (*p* = 0.003). Additionally, serum ferritin levels in cord blood in the GDM group were significantly higher than those in the non-GDM group (*p* = 0.003). The average age of patients at diagnosis was 29 years (range, 23–37 years). The mean gestational weeks of cases was 38 weeks. Maternal blood Hb, cord blood Hb, cord blood sTfR and maternal SF levels were not significantly different between the GDM group and corresponding control group, respectively (*p* = 0.391, *p* = 182, *p* = 0.364, *p* = 0.118, as shown in Table 2).

### 3.3. Expression of FPN, Hep, and TfR1 in the Placenta

**Figure 1:**
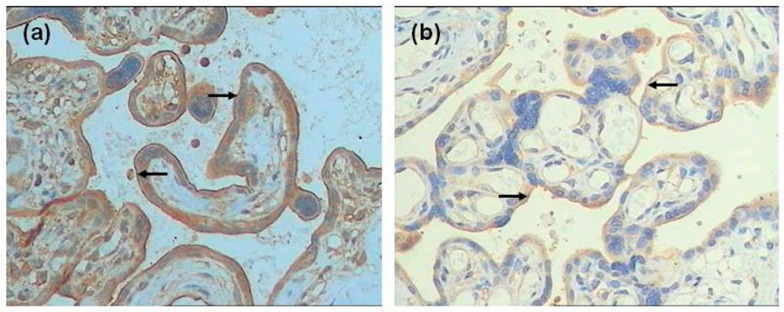

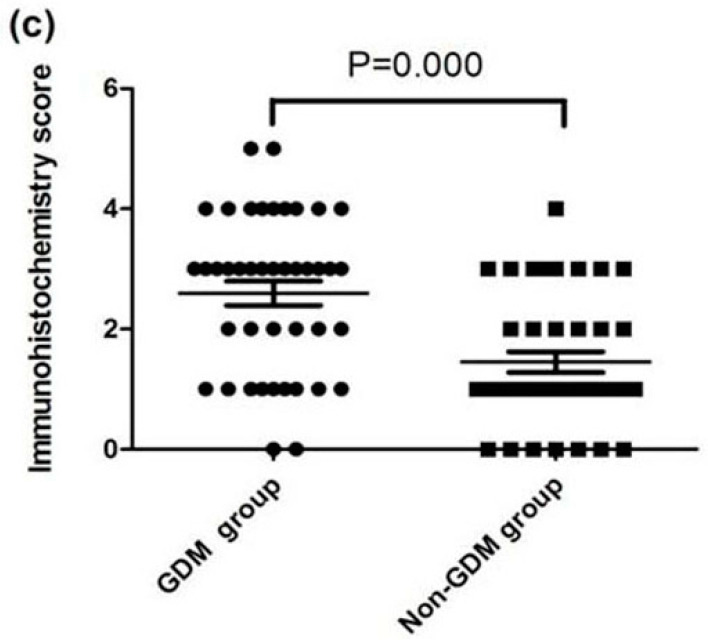
Immunohistochemistry images demonstrated that FPN1 was higher expressed on the membrane of STB cells of fetal surface(as arrows) from the GDM group (**a**) than that in non-GDM group (**b**, as arrows) (×200 magnification); Immunohistochemistry score was higher in the GDM group compared with the non-GDM group (**c**, *p* = 0.000).

**Figure 2:**
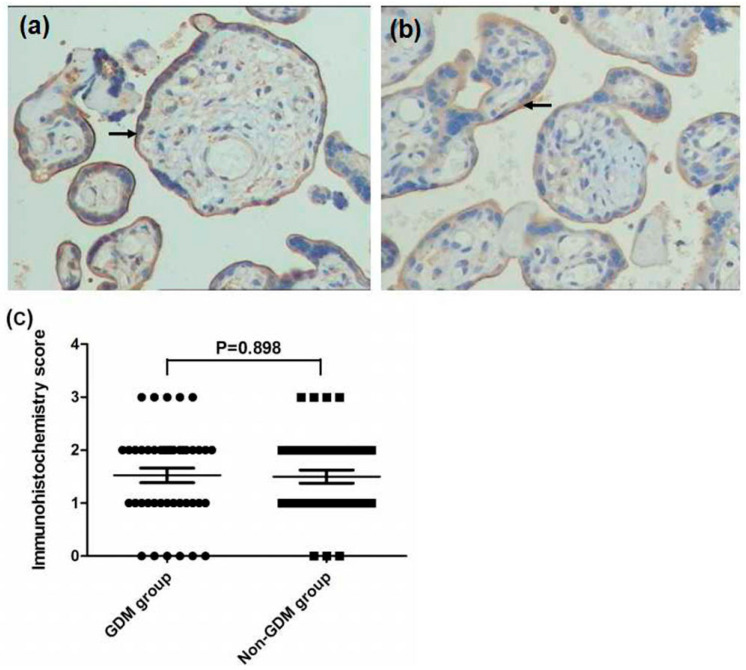
Immunohistochemistry images demonstrated that TfR1 was expressed on the membrane of maternal surface (as arrows) and there were no significant difference being found between the GDM group (**a**) and non-GDM group (**b**) (×200 magnification). No significant difference were foundin immunohistochemistry score between the two groups (**c**, *p* = 0.898).

**Figure 3:**
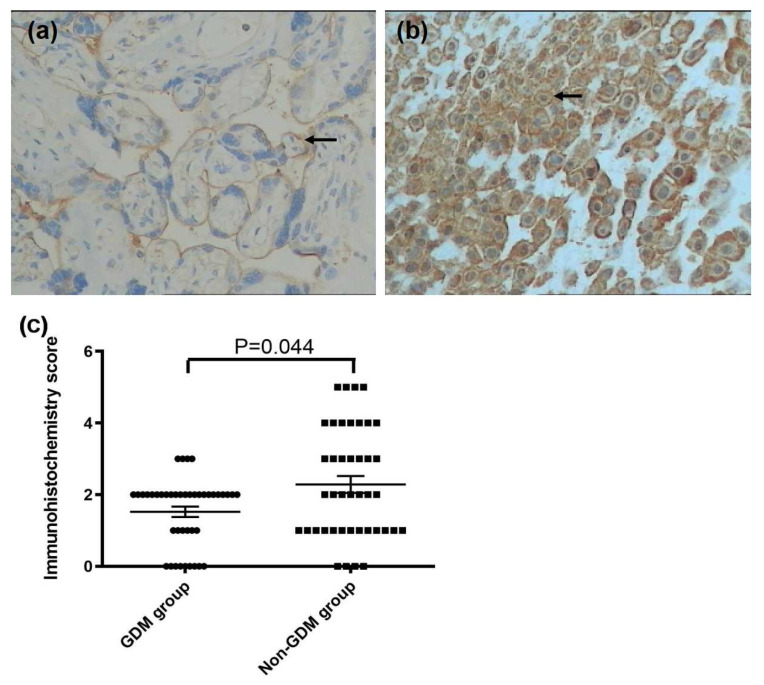
Immunohistochemistry images demonstrated that the lower expression of Hep on the membrane of the basal cells (arrow) in the GDM group (**a**) compared to that in the non-GDM group (**b**) (×200 magnification). A lower immunohistochemistry score was found in the GDM group compared with the non-GDM group (**c**) *p* = 0.044.

In placental tissue, significantly higher FPN expression and lower Hep expression were found in the GDM group compared with the non-GDM group (*p* = 0.000 and *p* = 0.044, respectively). There was no significant difference in TfR1 expression between the GDM group and control group (*p* = 0.898, as Figure 1, Figure 2 and Figure 3).

## 4. Discussion

In the present study, serum sTfR and ferritin levels of pregnant women and their newborns were measured to clarify the role of iron metabolism in maternal-fetal iron transfer and homeostasis under GDM. Our results provide further insight into the physiology of iron metabolism during pregnancy. Ferritin is thought to reflect body iron stores in healthy people and can predict development of type 2 diabetes [3,18]. Moreover, several studies have shown that increased iron stores in the general population is accompanied by an elevated incidence of diabetes [19,20]. Recent studies have also shown that pregnant women who develop GDM have higher levels of serum ferritin or Hb than women who do not develop GDM [20,21,22]. In our study, maternal serum ferritin and blood Hb levels were not significantly different in the GDM group compared with the non-GDM group at term pregnancy. Further, cord blood sTfR levels were similar between the GDM group and the non-GDM group. However, serum cord blood ferritin levels in the GDM group were significantly higher than those in the non-GDM group. Additionally, maternal sTfR levels in the GDM group were significantly higher than those in the non-GDM group. Fuchs and Ellinger reported that the human placenta is of the hemochorial type, whereby the fetally derived STB layer is bathed with maternal blood [23]. Therefore, cord blood ferritin is derived from maternal blood. Our results suggested that women with GDM increased iron transport to meet the augmented fetal iron demand in late pregnancy. During pregnancy, physiological iron demands are increased by maternal and fetal needs [24]. Some studies have shown that the growing fetus accumulates iron, particularly during late pregnancy, which increases the daily iron consumption requirements of the pregnant mother [25,26]. Iron transfer to the fetus becomes the priority over maternal requirements in late pregnancy. Therefore, maternal iron storage is depleted during the last trimester, while fetal iron levels are generally replete, unless the mother is severely iron deficient [27]. The current study suggests that enhancing iron transport from pregnant women with GDM to the fetus may increase the level of iron storage in their cord blood in late pregnancy.

Our results differed from those of data reported with regard to serum ferritin levels in GDM studies [4,5,28]. Some epidemiological studies have reported higher ferritin levels in women with GDM [4,5,28]. There is increasing recognition that GDM is an inflammatory condition that involves unbalanced inflammatory cytokine production [4,5]. Elevated serum ferritin levels, which have been reported in women with GDM in several studies [4,11,21,22], is possibly a result of an immune response by inflammatory cytokines, rather than adequate iron storage. The subjects in our study were not anemic, and iron therapy was not administered before the study (oral multivitamin being not excluded) There were also no differences in maternal anthropometry, Hb levels, or red cell indices at term pregnancy between the groups. SF levels at term pregnancy in women with GDM appear to serve as an iron source to meet the rapid need of fetus and to rapidly respond to an immune response. Therefore, SF levels are not an appropriate laboratory indicator for estimation of iron stores in an inflammatory state. However, sTfR levels can be used to reliably evaluate the degree of iron supply in GDM. Cellular iron metabolism and sTfR are proposed as a novel marker of iron status that is less affected by the presence of inflammation. Inconsistencies in serum ferritin results between studies may be explained by the fact that the current study was conducted at pregnancy term, unlike other similar previous studies that were conducted at mid-pregnancy. Moreover, SF levels are at maximum at 12–16 weeks of gestation and then fall with advancing gestation from hemodilution and mobilization of iron stores [29].

The present study showed that tissue Hep and FPN reflected functional iron status. Regulation of placental iron transfer is not well characterized. The placental STB expresses TfR1 and FPN [25,28,30]. Consistent with previous results, we also found that TfR1 was expressed on the maternal surface and FPN was expressed on the fetal surface of the placenta [31]. TfR1 functions to remove iron from the maternal circulation and deliver it to the fetal stroma. We also found that Hep was expressed in the basal decidua of the maternal-facing membrane and in the STB layer of the basal fetal-facing membrane. Additionally, the GDM group showed greater FPN staining intensity and lower Hep expression than did the non-GDM group. There was no significant difference in TfR1 expression between the two groups. These findings suggest that low expression of Hep leads to release of FPN in the STB layer, thereby increasing iron efflux into the fetal circulation to fulfill iron needs to the developing fetus at the final stage. However, excess iron is reserved in the form of ferritin to protect the fetus from iron overload, which is consistent with our result of cord blood SF levels.

Our study has several limitations. We were unable to measure serum insulin levels and serum hepcidin levels, which would have helped us to interpret the association with iron metabolism proteins, as well as the relationship between serum ferritin and insulin action or secretion. Another limitation is the lack of assay standardization for sTfR. Quantification of sTfR in the current study was obtained by ELISA, and values obtained from different methods, such as immunoturbidimetry, may be not comparable.

## 5. Conclusions

Taken together, our results indicate that pregnant women with GDM transport iron more actively than those without GDM. Different stages of gestation represent a continuum, always in a state of dynamic change. A condition characterized by chronic fetal hyperinsulinemia and hypoxia in pregnant women with GDM may play a role in fetal/placental iron demand and in the overall outcome of pregnancy. Further studies combining CRP, serum hepcidin, insulin or newborn blood glucose are clearly required to shed more light on this important issue.

## Figures and Tables

**Table 1:** Clinical characteristics of the studied populations.

Clinical Features	GDM (*n* = 42)	Non GDM (*n* = 40)	*p* Value
Maternal age (year)	30.52 ± 3.81	29.39 ± 3.76	0.090
BMI *	27.10 ± 2.39	25.96 ± 2.07	0.032
Newborn birth weight (kg)	3.25 ± 0.39	3.10 ± 0.34	0.083
Wk of gestation (week)	38.71 ± 1.0	37.70 ± 2.86	0.071
OGTT fasting (mg/dL)			
1-h *	10.30 ± 1.11	8.92 ± 0.94	0.000
2-h *	9.21 ± 1.22	7.71 ± 0.69	0.000

Results are mean ± SD. * *p* < 0.05. OGTT fasting, a 2-h, 75-g oral glucose tolerance test; BMI, body mass index.

**Table 2:** Comparison of iron metabolism indicators in the maternal–fetal interface with and without GDM.

Group	GDM (*n* = 42)	Control (*n* = 40)	*p* Value
Maternal Hb (g/dL)	116.20 ± 7.0	117.76 ± 8.31	0.391
Umbilical cord Hb (g/dL)	158.14 ± 9.54	154.76 ± 11.51	0.182
Maternal blood sTfR (nM)	31.82 ± 11.51	22.80 ± 8.30	0.003 ^a^
Cord blood sTfR (nM)	34.63 ± 8.33	32.60 ± 6.00	0.364
Maternal SF (ng/mL)	21.71 (13.86–42.62) ^Δ^	30.90 (25.00–39.00) ^Δ^	0.118
Umbilical cord SF (ng/mL)	172.75 (112.08–192.03) ^Δ^	111.6 (68.29–150.95) ^Δ^	0.003 ^b^

sTfR, soluble transferrin receptor; SF, serum ferritin; Data were shown as mean ± SD unless otherwise specified; ^Δ^ data are expressed as median and percentile values(P25-P75); ^a^ Indicate *p* < 0.01 as compared to control as assessed by *t*-test; ^b^ Indicate *p* < 0.01 as compared to control as assessed by Man-Whitney test.

## Abbreviations

The following abbreviation are used in this manuscript:

GDM	Gestational Diabetes
STfR	Soluble transferrin receptor (sTfR)
SF	Serum ferritin
FPN	Ferroportin
NGT	Normal glucose tolerance
STB	Syncytiotrophoblast
HCT	Hematocrit
OGTT	Oral glucose tolerance test
HEP	Hepcidin
