# Correction: Yang et al. Expression of Hepcidin and Ferroportin in the Placenta, and Ferritin and Transferrin Receptor 1 Levels in Maternal and Umbilical Cord Blood in Pregnant Women with and without Gestational Diabetes. *Int. J. Environ. Res. Public Health* 2016, *13*, 766

**DOI:** 10.3390/ijerph22040636

**Published:** 2025-04-18

**Authors:** Anqiang Yang, Jun Zhao, Minhua Lu, Ying Gu, Yunlong Zhu, Daozhen Chen, Jinyan Fu

**Affiliations:** 1 Department of Pathology, Wuxi Maternity and Child Health Hospital Affiliated to Nanjing Medical University, Wuxi 214002, China; yaq1234@126.com (A.Y.); mhlu2005@sina.com (M.L.); 2 Clinical Laboratory, Wuxi Maternity and Child Health Hospital Affiliated to Nanjing Medical University, Wuxi 214002, China; chalange@163.com (J.Z.); chendaozhen@163.com (D.C.); 3 Department of Obstetrics, Wuxi Maternity and Child Health Hospital Affiliated to Nanjing Medical University, Wuxi 214002, China; 18914137366@189.cn (Y.G.); sequoia113847@163.com (Y.Z.)

In the original publication [1], there was a mistake in Figure 3c as published. In referring to the description for Hepcidin expression (as Figure 3c), the order of priority for the GDM group and the non-GDM group was reversed in the original version and in the revised version. The authors wish to apply for a correction. Figure 3c should be replaced with the following figure: 

The authors apologize for any inconvenience caused and state that the scientific conclusions are unaffected. This correction was approved by the Academic Editor. The original publication has also been updated. 

## Figures and Tables

**Figure 3:**
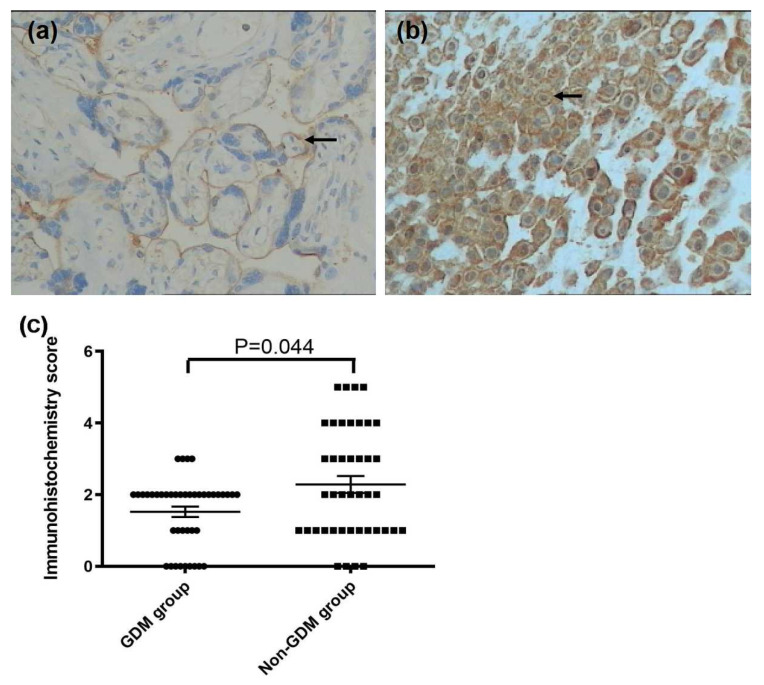
Immunohistochemistry images demonstrated that the lower expression of Hep on the membrane of the basal cells (arrow) in the GDM group (**a**) compared to that in the non-GDM group (**b**) (×200 magnification). A lower immunohistochemistry score was found in the GDM group compared with the non-GDM group (**c**) *p* = 0.044.
